# Risk stratification for post‐operative pulmonary complications following major cardiothoracic or abdominal surgery: Validation of the PPC Risk Prediction Score for physiotherapist's clinical decision‐making

**DOI:** 10.1111/crj.13579

**Published:** 2023-01-03

**Authors:** Sofie Langbo Salling, Janne Hastrup Jensen, Sebastian Breddam Mosegaard, Lotte Sørensen, Inger Mechlenburg

**Affiliations:** ^1^ Department of Clinical Medicine Aarhus University Aarhus Denmark; ^2^ Department of Orthopaedics Aarhus University Hospital Aarhus Denmark; ^3^ Department of Physiotherapy and Occupational Therapy Aarhus University Hospital Aarhus Denmark; ^4^ Department of Public Health Aarhus University Aarhus Denmark

**Keywords:** general surgery, lung, pneumonia, post‐operative complication, risk factors, validation studies as topic

## Abstract

**Introduction:**

Patients undergoing major cardiothoracic or abdominal surgery are at increased risk of developing post‐operative pulmonary complications (PPC), but respiratory physiotherapy can prevent PPC. We have previously developed the PPC Risk Prediction Score to allocate physiotherapists' resources and stratify patients into three risk groups. In this study, we performed a temporal external validation of the PPC Risk Prediction Score. *Such validation is rare and adds to the originality of this study*.

**Methods:**

A cohort of 360 patients, admitted to undergo elective cardiothoracic or abdominal surgery, were included. Performance and clinical usefulness of the PPC Risk Prediction Score were estimated through discrimination, calibration and clinical usefulness, and the score was updated.

**Results:**

The score showed c‐statistics of 0.62. Related to clinical usefulness, a cut point at 8 gave a sensitivity of 0.49 and a specificity of 0.70, whereas a cut point at 12 gave a sensitivity of 0.13 and a specificity of 0.95. Two predictors included in the development sample score, thoraco‐abdominal incision odds ratio (OR) 2.74 (1.12;6.71) and sternotomy OR 2.09 (1.18;3.72), were statistically significantly associated to PPC in the validation sample.

**Conclusions:**

The score was not able to discriminate between patients with and without PPC; neither was the updated score, but the study identified clinically relevant risk factors for developing PPC.

## Graphical abstract

This study describes a validation of a risk prediction score for post‐operative pulmonary complications (PPC) following major cardiothoracic or abdominal surgery. The score was not able to discriminate between patients with and without PPC, but the study identified clinically relevant risk factors for developing PPC.

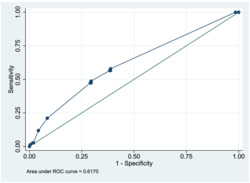

## 1 INTRODUCTION

Post‐operative pulmonary complications (PPC) are common adverse events in patients undergoing major surgery.^1^ The estimated incidence of PPC is 1%–35% depending on differences in PPC definition, patient population and type of surgical intervention.^2^, ^3^, ^4^

Depending on severity, PPC may have significant effect on mortality, morbidity, intensive care unit admission, length of hospitalization and healthcare costs.^1^, ^3^, ^5^ Thus, lowering the risk of PPC is of great interest to both patients and the healthcare system.

In recent years, a tendency to use minimal invasive surgeries has gained ground,^6^ and patients with a higher comorbidity burden and a potentially higher risk of developing PPC can now be offered surgical treatment.

Respiratory physiotherapy is, among other initiatives as pain management, anesthetic techniques and intra‐operational techniques, the first‐line treatment to prevent PPC.^3^, ^7^ Patients undergoing major cardiothoracic or abdominal surgery are offered respiratory physiotherapy as standard care,^8^ resulting in extensive use of physiotherapy department resources at hospitals delivering highly specialized surgery. This has given rise to a request to develop a risk prediction score to assist physiotherapists in the allocation of respiratory physiotherapy resources for patients undergoing major cardiothoracic or abdominal surgery.^9^

Likewise, the use of risk prediction scores is broadly supported by healthcare providers and policymakers to assist clinical decision‐making and allocation of resources to improve cost‐effectiveness and individualized patient care.^10^, ^11^, ^12^ Numerous risk prediction scores for PPC have previously been developed,^1^, ^7^, ^13^, ^14^, ^15^ but no prediction score has to our knowledge been developed and externally validated in a cohort of patients undergoing major cardiothoracic or abdominal surgery, where preventive respiratory physiotherapy is part of the standard care.

A Delphi process and a cohort of 339 patients formed the basis for the development of the PPC Risk Prediction Score in accordance with the recommended standards from the PROGnosis RESearch Strategy Partnership (PROGRESS) and the Transparent Reporting of a multivariable prediction model for Individual Prognosis Or Diagnosis (TRIPOD) guidelines.^4^, ^10^, ^16^ This work resulted in a prediction score identifying four independent predictors of PPC.^4^

Importantly, before implementing the prediction score in clinical practice, it is necessary to externally validate the score to clarify if it provides valid predictions in other patient samples than the sample used for the model development.^11^

Hence, the aim of this study was to externally validate if the PPC Risk Prediction Score^4^ was able to discriminate between low, moderate and high risk of PPC in a new sample of patients undergoing major cardiothoracic or abdominal surgery.

## 2 MATERIALS AND METHODS

The study is a prospective temporal validation of the PPC Risk Prediction Score performed in accordance with guidelines from the (TRIPOD) Explanation and Elaboration^16^ and the PROGRESS framework.^10^

### 2.1 The PPC Risk Prediction Score

The newly developed PPC Risk Prediction Score for patients undergoing major cardiothoracic or abdominal surgery identified four independent predictions of PPC: reduced lung function [forced expiratory volume in 1st second (FEV_1_) ≤ 50% of predicted and FEV_1_ > 50% < 75% of predicted], unintended weight loss within the last 3 months >10 kg, sternotomy and thoraco‐abdominal incision. Based on assigned point values, patients were divided into three risk groups: <9 points, low risk; 9–15 points, moderate risk; and >15 points, high risk (Table 1).

**TABLE 1:** PPC Risk Prediction Score point values^1^

	Assigned point values
Patient‐related predictors	
Reduced lung function	
FEV_1_ > 50% < 75% predicted	9
FEV_1_ ≤ 50% predicted	16
Unintended weight loss >10 kg	15
Surgical‐related predictors	
Sternotomy	13
Thoraco‐abdominal incision	16

*Note*: Three levels of risk were indicated by the following cut‐offs 9 < points, low risk; 9–15 points, moderate risk; and >15 points, high risk.

Abbreviations: FEV_1_, forced expiratory volume in 1st second; PPC, post‐operative pulmonary complications.

### 2.2 Study design and setting

Patients undergoing major cardiothoracic or abdominal surgery at Aarhus University Hospital, Denmark, included from September 2020 to February 2021 formed the basis for this temporal external validation of the PPC Risk Prediction Score.

### 2.3 Participants

Patients were eligible for inclusion, if they were scheduled for elective open surgery or thoracoscopy procedures involving cardiac, aortic, gastrointestinal, urological or pulmonary organs, performed in general anesthesia and with mechanical ventilation.

Primary exclusion criteria were age below 18 years old and being unable to give written informed consent to study participation. Secondary exclusion criteria were pre‐operative pneumonia, cancelled surgery not rescheduled within the study period, unexpected post‐operative intubation exceeding 24 h due to non‐pulmonary complications, two or more re‐surgeries during admission or post‐operative non‐pulmonary complications and additional surgical procedures affecting the prognosis for PPC development within 30 days post‐operatively. Exclusion of patients was made by two research physiotherapists and cases of doubt were resolved by consulting a third research colleague.

### 2.4 Standard respiratory physiotherapy

All patients were seen for a pre‐operative physiotherapy session, including guidance in airway clearance techniques and information about potential respiratory complications in relation to surgery.

Following surgery, all patients received standard post‐operative respiratory physiotherapy until ambulation and airway clearance techniques could be maintained independently and oxygen saturation was close to habitual.

Standard post‐operative care consisted of information and instructions in PPC‐preventive strategies such as mobilization and deep breathing exercises using a positive expiratory pressure device (PEP) with mouthpiece (Intersurgical); the usage of PEP was encouraged every second waking hour with three sets of 10 repeated breathings each.

If necessary, more frequent sessions and intermittent continuous positive airway pressure (CPAP) (Lövenstein or Whisperflo) with a mask covering mouth and nose were provided. If provided, CPAP was given with the support of a physiotherapist or by the patients themselves two or three times a day with a standard treatment consisting of 25–30 breathings repeated for three or four sets.

### 2.5 Data collection

Pre‐operative data were collected from five research physiotherapists through patient anamnesis, medical records and physical assessments, after signed informed consent was provided. When patients were seen for their scheduled pre‐operative physiotherapy session, a thorough description on identification and collection of pre‐operative variables from the medical record and patient anamnesis was followed.

In addition to the potential predictors collected in the development study, three new potential predictors, the New Mobility Score,^17^ 30‐Second Chair Stand Test^18^ and pre‐operative oxygen saturation (SpO2), were collected with the purpose of updating the score to improve performance and clinical usefulness.

Self‐reported data were collected on lifestyle conditions, comorbidities and patient factors, including habitual productive cough, repeated lung infections within the last year, reduced strength of cough as a result of neurological illness, active smoking (in the last 48 h), end of smoking within the last 4 weeks, pre‐operative chemotherapy or radiation therapy, unintended weight loss within the last 3 months (kg), difficulty swallowing, height (cm), weight (kg), The New Mobility Score, age, sex and chronic heart failure (NYHA≥3).

Lung function (FEV_1_) assessment using a handheld spirometer (Vitalograph® Micro), 30‐Second Chair Stand Test^18^ and pre‐operative oxygen saturation (SpO2) were measured by the research physiotherapists according to recommendations, when they were seen at the scheduled pre‐operative physiotherapy session.

**FIGURE 1:**
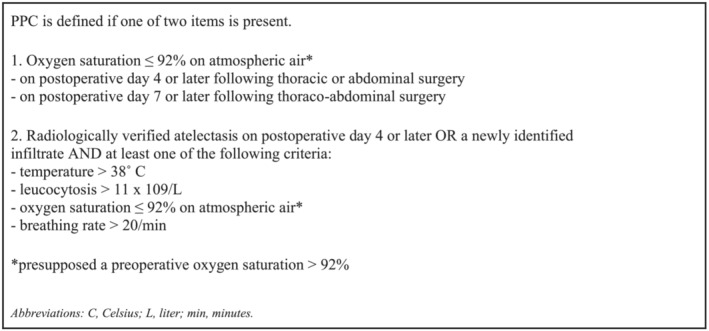
Definition of post‐operative pulmonary complications (PPC) (Delphi panel 2015).

Primary outcome was PPC occurring within 30 days post‐operatively, referring to the definition developed by a Delphi panel for the purpose of the PPC Risk Prediction Score^4^ (Figure 1). The outcome was considered a binary categorical variable (yes/no) (Figure 1). To identify PPC, post‐operative oxygen saturation was measured by the research physiotherapist at Day 4 or later in patients treated with thoracic or abdominal surgery and at Day 7 or later in patients treated with thoraco‐abdominal surgery. Additional information necessary to identify PPC such as temperature >38°C, leukocytosis >11 × 10^9^/L and breathing rate >20/min was retrieved from the medical record.

Post‐operatively, data were collected from the medical record on surgical procedure, length of hospitalization, rehospitalization within the first 30 post‐operative days, mortality within the first 30 post‐operative days and extended post‐operative physiotherapy interventions.

### 2.6 Blinding

Research physiotherapists, blinded to the four predictors described in the PPC Risk Prediction Score, obtained informed consent, collected pre‐operative patient data and performed physical tests at the standard pre‐operative care consultation. Post‐operative data were collected from medical records by research physiotherapists, blinded to pre‐operative data. Doubts about primary outcome were consulted with a research physiotherapist colleague, and if agreement was not achieved, a third research physiotherapist was consulted.

### 2.7 Statistical analysis

#### 2.7.1 Power

The sample size was estimated according to the recommendations in the TRIPOD guidelines and Vergouwe et al, where 100 events and 100 non‐events in the sample are needed to validate a prediction model.^12^, ^19^

#### 2.7.2 Missing data and extreme values

Patients with missing primary outcome data were excluded from the analysis. When patients had missing potential predictors, we performed mean or median imputation to ensure complete case analysis for the logistic regression modelling.^12^

#### 2.7.3 Potential predictors

PPC incidence was calculated and associations between development of PPC and potential predictors were assessed. Comparative analyses of demographics and potential predictors between PPC Risk Prediction Score and the validation sample were performed for patients with and without PPC. Continuous data were presented with mean and standard deviations or with median and interquartile ranges. Differences between patients with and without PPC were tested using the unpaired *t*‐test or the Wilcoxon signed‐rank test. Categorical data were presented numerically and analyzed using Chi^2^ test for binary variables and test for trend for categorical data with more than two exposure levels to compare patients with and without PPC.

#### 2.7.4 Model performance

Model performance of the PPC Risk Prediction Score in the validation sample was studied through discrimination, calibration and clinical usefulness. We estimated the model's ability to discriminate between patients with and without the outcome using c‐statistics and graphic illustration by a receiver operating characteristics curve (ROC). The calibration was described by comparing the predicted and observed number of events and illustrated by a calibration slope.

The clinical usefulness of the score was assessed by means of measures of accuracy of outcome diagnosis. Also, we re‐estimated all four regression coefficients included in the PPC Risk Prediction Score in the validation study through a logistic regression model. We used a bootstrap method deriving 500 computer‐generated subsamples by random selection with replacement.^12^

A bootstrap‐corrected β coefficient for each predictor in the updated logistic regression model was calculated, and a risk score was assessed for each predictor by assigning points driven by multiplying the regression coefficients by 10 and rounding to the nearest integer. Finally, three risk groups were defined based on thresholds from the cumulated risk score distribution. These measures of PPC were analyzed for high‐risk scores compared with moderate‐risk and low‐risk scores, respectively.

We attempted to update the logistic regression model with various combinations and were continuously testing the model performance. Predictors were removed sequentially based on a 0.05 significance level.

All statistical analyses were performed using STATA, Version 17.0 IC (StataCorp, College Station, TX, USA).

## 3 RESULTS AND DISCUSSION

PPC was recorded for 76 out of the 334 patients (22.8%) within the 30‐day follow‐up period, which was lower than in the development sample (33.3%).

In the validation sample, 30‐day in‐hospital mortality was significantly higher in patients with PPC than in patients with no PPC (*p* < 0.05). The same applied to post‐operative length of stay in days, 11 versus 7 (*p* < 0.001) (Table 2).

**TABLE 2:** Demographic and clinical characteristics of PPC Risk Prediction Score validation sample (*n* = 334)

	Total	No PPC (*n* = 258)	PPC (*n* = 76)	*P* value
Gender, *n* (%)				
Female	125(37.4)	103(40)	22(28.9)	
Male	209(62.6)	155(60)	54(71.1)	0.082
Age				
>80 years, *n* (%)	14(4.2)	‐	<5	
≤80 years	320(95.8)	‐	‐	>0.05
Current smoking, *n* (%)				
Yes	42(12.6)	28(10.9)	14(18.4)	
No	292(87.4)	230(89.1)	62(81.6)	0.08
Chronic productive cough, *n* (%)				
Yes	101(30.2)	77(29.8)	24(31.6)	
No	233(69.8)	181(70.2)	52(68.4)	0.772
Repeated lung infections, *n* (%)				
Yes	<5 (1.2)	<5	<5	
No	330(98.8)	‐	‐	<0.05*
Pre‐operative spirometry, *n* (%)				
FEV_1_ ≥ 75% pre	279(83.5)	219(84.9)	60(78.9)	
FEV_1_ > 50 to < 75% pre	49(14.7)	‐	‐	
FEV_1_ < 50% pre	6(1.8)	<5	<5	>0.05
Pre‐operative oxygen saturation (SpO2) (IQR) range 5%–95%		*n* = 258	*n* = 76	
		98 (1) 95 to 100	97.5 (1.5) 95 to 99	0.098
Impaired swallowing, *n* (%)				
Yes	26(7.8)	21(8.1)	5(6.6)	
No	308(92.2)	237(91.9)	71(93.4)	0.655
Insufficient cough from neurological disease, *n* (%)				
Yes	333(99.7)	<5	<5	
No	1(0.3)	‐	‐	>0.05
Chronic heart failure (NYHA >3), *n* (%)				
Yes	0 (0)	0	0	
No	334 (100)	258(100)	76(100)	
Unintended weight loss within the last 3 months >10 kg, *n* (%)				
Yes	5(1.5)	<5	<5	
No	329(98.5)	‐	‐	>0.05
Chemotherapy, *n* (%)				
Ye	37(11.1)	29(11.2)	8(10.5)	
No	297(88.9)	229(88.8)	68(89.5)	0.862
New mobility score (NMS), *n* (%) > 6				
Yes	8(2.4)	‐	<5	
No	326(97.6)	‐	‐	>0.05
30‐second sit to stand test (30CTS), median (IQR) range 5%–95%		*n* = 258	*n* = 76	
		13 (5)8 to 20	12(3)6 to 19	0.136
Surgical incision, *n* (%)				
Thoracotomy	22(6.6)	14(5.5)	8(10.5)	0.115
Laparotomy (aortic)	20(6.0)	9(3.5)	11(14.5)	<0.001*
Laparotomy (gastrointestinal)	58(17.5)	49(19.2)	9(11.8)	0.148
Laparotomy (urological)	31(9.4)	‐	<5	<0.005**
Sternotomy	87(26.3)	60(23.5)	27(35.5)	0.032*
Hemisternotomy	19(5.7)	‐	<5	0.190
Thoraco‐abdominal incision	26(78.5)	16(6.3)	10(13.2)	0.047*
Video‐assisted thoracoscopy	64(19.3)	57(22.4)	7(9.2)	0.012**
Other	4(1.2)	<5	<5	0.914
Extended respiratory physiotherapy, *n* (%)				
Yes	57(17.5)	17(6.8)	40(54.1)	
No	268(82.5)	234(93.2)	34(45.9)	<0.001*
Post‐operative length of stay in days, median (IQR) range 5%–95%		*n* = 255	*n* = 71	
		7(4)3 to 17	11(7)5 to 23	<0.001*
30‐day hospital readmission, *n* (%)				
Yes	64(19.3)	45(17.9)	19(25.7)	
No	261(78.6)	206(82.1)	55(74.3)	0.141
30‐day in‐hospital mortality, *n* (%)				
Yes	<5	<5	<5	
No	328	‐	‐	<0.05*

*Note*: *statistically significant difference, **statistical significant difference with a protective effect, − removed data due to General Data Protection Regulation <5 patients.

Abbreviations: FEV1, forced expiratory volume in the first second; IQR, interquartile range; n, number; NYHA, New York Heart Association Functional Classification; PPC, post‐operative pulmonary complications; SD, standard deviation, VATS, video‐assisted thoracoscopic surgery.

### 3.1 Study population

A total of 409 eligible patients were identified during the study period. Of these, 360 patients were assessed for inclusion as 35 eligible patients were not recruited due to unavailability of the research physiotherapist and 14 patients declined to participate.

**FIGURE 2:**
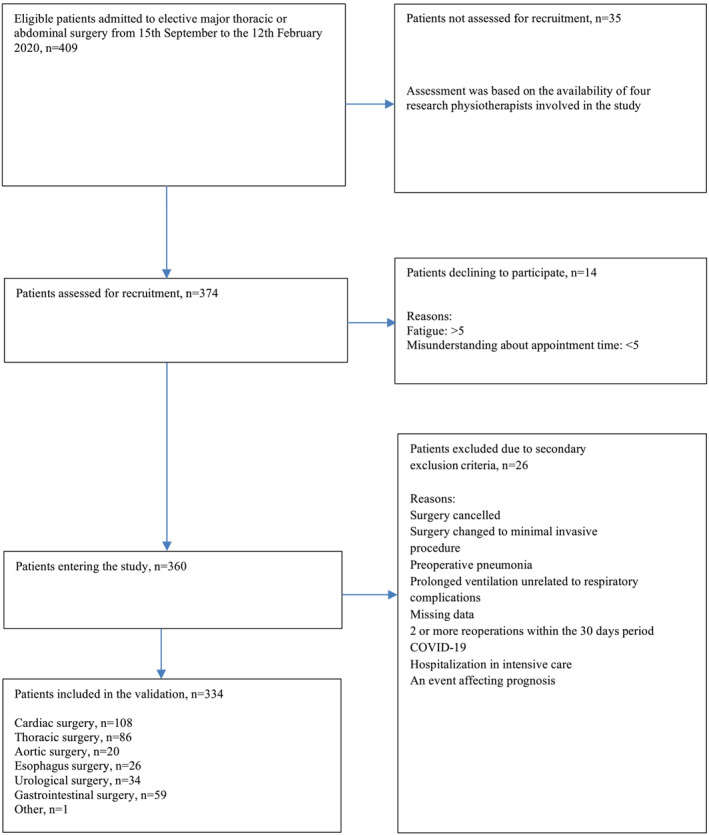
Flowchart of patient recruitment at Aarhus University Hospital (*n* = 409)

Due to secondary exclusion criteria 26 patients were excluded, which allowed 334 patients to be included in the study (Figure 2).

Mean imputation of lung function (FEV_1_) was performed in two patients with missing data. Median imputation of data was performed with seven patients with missing data on the 30‐Second Chair Stand Test and three patients with missing data in the New Mobility Score.

Fifteen patients had missing data on predictors included in the PPC Risk Prediction Score or in new potential predictors. With regard to the predictors included in the score, patients were representative.

### 3.2 Demographics and comparison with the PPC Risk Prediction Score data of the distribution of predictors

The distribution of surgical incisions and events of predictors differed between the development sample^4^ and the validation sample (Table 2).

Some statistically significant associations from the univariate analysis in the development sample were reproduced in the validation sample: repeated lung infections within the last year, sternotomy and thoraco‐abdominal incision were all predictors statistically significantly associated with a higher incidence of PPC in both the development sample^4^ and the validation sample (Table 2).

Additionally, video‐assisted thoracoscopic surgery (VATS) was statistically significantly associated with a lower incidence of PPC in both the development sample^4^ and the validation sample (Table 2).

### 3.3 Model performance

**FIGURE 3:**
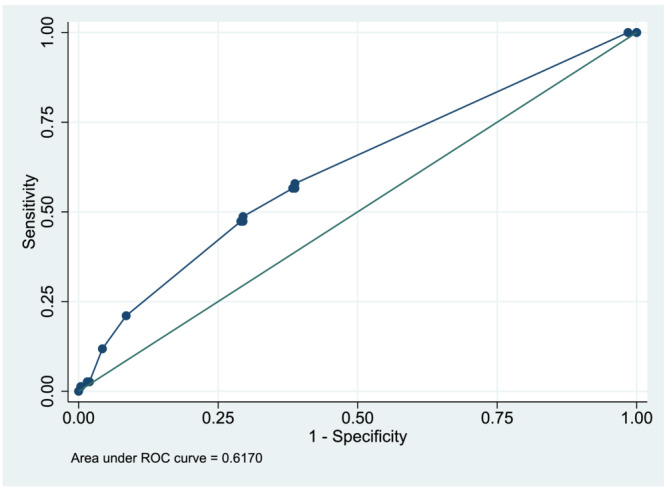
Receiver operating characteristics (ROC) curve to show discrimination of the post‐operative pulmonary complications (PPC) risk prediction score in the validation sample.

When estimating performance of the logistic regression model, we found a discrimination of 0.62 (Figure 3). When re‐estimating the assigned point value for each predictor, unintended weight loss within the last 3 months odds ratio (OR) 1.36(0.19;9.7), FEV_1_ > 50% < 75% of predicted OR 1.51(0.75;3.02), thoraco‐abdominal incision OR 2.74(1.12;6.71) and sternotomy OR 2.10(1.18;3.72) all showed an increased risk of PPC. The assigned point value for lung function FEV_1_ ≤ 50% of predicted OR 0.70(0.08;6.31) was equal to no increased risk of PPC (Table 3).

**TABLE 3:** Independent predictors of risk for post‐operative pulmonary complications identified by multiple logistic regression analysis and corrected by bootstrap analysis (*n* = 334)

	Multivariable analysis (PPC *n* = 76)		Corrected ß‐coefficients	Assigned point values
	OR (95% CI)	ß‐coefficients (95% CI)		
Patient‐related predictors				
Reduced lung function				
FEV_1_ ≥ 75% predicted	1			
FEV_1_ > 50% < 75% predicted	1.51(0.75;3.02)	0.41(−0.64;1.02)	0.436	4
FEV_1_ ≤ 50% predicted	0.70(0.08;6.31)	−0.36(−1.43;0.46)	−0.004	0
Unintended weight loss within the last 3 months >10 kg	1.36(0.19;9.7)	0.31(−1.87;2.06)	0.408	4
Surgical‐related predictors				
Sternotomy	2.10(1.18;3.72)	0.74(0.11;1.34)	0.765	8
Thoraco‐abdominal incision	2.74(1.12;6.71)	1.01(0.00;1.95)	1.114	11
Model intercept	0.20(0.14;0.29)	−1.60(−1.99;‐1.23)	−1.635	

*Note*: Two levels of risk were indicated by the following cut‐offs: <8 points, low‐ to moderate risk, and ≥8 points, high risk.

Abbreviations: FEV1, Forced expiratory volume in 1st second; OR, odds ratio; CI, confidence interval; PPC, post‐operative pulmonary complications; β, beta.

**FIGURE 4:**
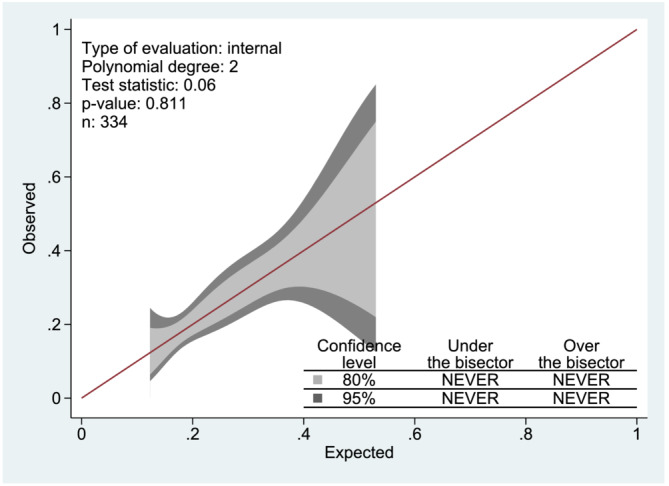
Calibration slope to show calibration of the post‐operative pulmonary complications (PPC) risk prediction score.

Cut‐offs for risk groups were based on the thresholds from the cumulated risk score distribution. Clinically reasonable cut‐offs were considered at 75th and 95th percentiles, for moderate and high risk, respectively. Hence, a cut‐off between low‐ and moderate‐risk group and between moderate and high‐risk group were set at cut‐off points 8 and 12, respectively. The predicted risk of developing PPC for each patient was between 17% and 52%. The predicted outcome against observed outcome is shown in a calibration slope (Figure 4).

A cut off at 8 points gave a sensitivity of 0.49 (49%) and a specificity of 0.70 (70%), and a cut off at 12 points gave a sensitivity of 0.13 (13%) and a specificity of 0.95 (95%) (Table 4).

**TABLE 4:** Proportion of PPC in the low, moderate‐ and high‐risk groups, *n* = 334

	Low risk	Moderate risk	High risk	Total
	<8 points	8–12	>12 points	
No PPC, *n*	*n* = 182	*n* = 64	*n* = 12	*n* = 258
Percentage	82.35% (true negative)	70.33%	54.55% (false positive)	77.25%
PPC, *n*	*n* = 39	*n* = 27	*n* = 10	*n* = 76
Percentage	17.65% (false negative)	29.67%	45.45% (true positive)	22.75%
Total	*n* = 221	*n* = 91	*n* = 22	*n* = 334

Abbreviations: *n*, number; PPC, post‐operative pulmonary complications.

### 3.4 Update of the model

Different combinations of predictors in the logistics regression model were tested to update the PPC Risk Prediction Score, including adding three new potential predictors.

However, a satisfactory discrimination between patients with and without PPC was still not achieved.^12^

### 3.5 Discussion

An external validation of a pre‐operative risk prediction score for PPC is rarely conducted, and the PPC Risk Prediction Score is to our knowledge the first temporal externally validated risk prediction score developed with a physiotherapy perspective to surgery patients receiving preventive respiratory physiotherapy as standard care, which adds new knowledge to the field.

We calculated the logistic regression model's ability to discriminate between patients with and without a PPC and found a discrimination value of c‐statistics of 0.62 in the validation sample. A satisfactory discrimination is often considered to be between 0.7 and 0.9,^20^ and it is to be expected that a model's predictive performance is poorer in the validation sample than in the sample in which the model was developed.^16^ The discrimination in the model development sample was only c‐statistic 0.7, which has been a determining factor in the poor discrimination value in this study. However, a model should be evaluated based not only on discrimination but also on the calibration and clinical usefulness to support clinical decision‐making.^21^

To evaluate the logistic regression model's ability to guide decision‐making, we made three risk prediction groups: a low‐, moderate‐ and a high‐risk group. Our score differentiated between risk levels of PPC, as 45% of patients in the high‐risk group and only 18% of patients in the low‐risk group developed a PPC (Table 4). If applying the score to clinical practice, this means that 55% of patients in high‐risk group may be categorized as false positive and 18% in low‐risk group as false negative. As the low‐risk group proportion (18%) is close to the incidence in the full sample (23%), the clinically usefulness of the score may therefore be minor.

In our sample, 49 out of 409 (12%) eligible patients were not included. Patients not assessed for recruitment were missing random, and the distribution is considered non‐differentiated to predictors and outcome.

When explaining model performance, sample size of the study must be considered to be an important contributing factor. Less than 10 patients had unintended weight loss >10 kg within the last 3 months or reduced lung function, FEV_1_ ≤ 50% of predicted, which may have influenced the estimate of the predictors and the model's performance further.^22^ The difference in effect of predictors between the development and validation sample can be explained by chance, or by overfitting of predictors in the original model.^12^, ^21^

Due to the incidence of PPC in the development sample (33%), we suggested that a sample size of 350 patients would generate about 100 events, as recommended in the TRIPOD guidelines. In our study, a prevalence of 23% left us with only 76 events. Our findings do not conflict with previous findings.^2^, ^3^ However, the sample size might have affected the internal validity of the study, because the number of events is considered an important factor when testing model performance in a new cohort of patients.^12^

Also, PPC event rate in the validation sample may be related to the surgical procedures, which are developing fast and changing to less invasive procedures with a shorter recovery over time. Likewise, the risk of misclassification of outcomes, as well as predictors, could possibly have affected the event rate in our study. We believe the risk of misclassification of PPC to be minimal because a second and third research physiotherapist was consulted in cases of doubt. Also, the Covid‐19 pandemic has been present during the data collection and may have affected the timing and number of patients undergoing surgery during our study period.

With no success, we attempted to update the PPC Risk Prediction Score by adding new potential predictors to the model. However, a risk prediction score for post‐operative pulmonary complication amendable to respiratory physiotherapy is still of relevance.

Although our findings showed minor clinical usefulness of the PPC Risk Prediction Model, we identified valuable information in the univariate analysis, in both the development sample and the validation sample.

In existing literature, surgical incision is found to be a risk factor for PPC^3^; comparable findings were registered in our study: Sternotomy and thoraco‐abdominal incision were statistically significantly associated with a higher incidence of PPC. In addition, video‐assisted thoracoscopy (VATS) was statistically significantly associated with a lower incidence of PPC. Our study and existing literature suggest that surgical incision is of interest to clinical practice and in the development of future prediction models for PPC.^3^, ^4^

Also, repeated lung infections within the last year were statistically significantly associated with PPC in both the development sample and the validation sample and might be of interest for further research.

When determining the poor performance of the model, different causes have challenged the development and validation of the model. We consider the development study to be of high methodologic quality, because it has been following the standards for developing risk prediction scores and has made a careful selection of risk factors.^4^ But the fact that all patients received respiratory physiotherapy and more than 20% of the patients in our study received extended respiratory physiotherapy may have weakened the discriminatory performance of the model. Presumably, more patients would have developed a PPC if only routine treatment had been provided but for ethical reasons this was not possible.

Furthermore, it is difficult to take into account intra‐operative and post‐operative events that might have been of relevance for development of PPC.

We only identified one prediction score for PPC with good performance in an external validation,^23^ although several scores have been developed.^15^ Through this study, we have likewise uncovered the complexity of the development of a score for PPC. In addition, time and development in operative techniques, post‐operative pain management and pre‐operative optimization of nutrition and physical function, contribute to the complexity when developing a score, which will not quickly become outdated. Also, making a risk prediction score on a wide group of patients undergoing different surgical procedures has turned out to be challenging, and future studies may benefit from focusing on limited patient groups.

Through this study, we have uncovered surgical procedures and patient factors, which are associated with an increased risk of developing a PPC. In addition, we have been aware of the necessity of individual pre‐ and post‐operative assessment from physiotherapists in combination with a valid risk prediction score.

We believe future studies should focus on continued research in developing and validating feasible tools and guidelines to help clinicians make evidence‐based decisions with regard to PPC. Adherence to the progress framework and TRIPOD guideline as well as ensuring large enough sample sizes is essential/recommended in future study planning.

## 4 CONCLUSION

The PPC Risk Prediction Score showed minor clinical usefulness. The score was able to identify 49% of the patients who would develop PPC by a cut‐off at 8 points. The model was not able to make a satisfactory risk stratification into low‐risk, moderate‐risk and high‐risk groups, even after re‐estimating and including new predictors into the model.

However, we found that thoraco‐abdominal incision and sternotomy were statistically significantly associated with PPC in the univariate analysis in both the development sample and the validation sample.

## CONFLICT OF INTEREST

The authors report no conflict of interest.

## ETHICS STATEMENT

The Central Jutland Region Committee on Biomedical Research Ethics concluded that the study was exempt from ethical notification and approval (Case No. 1‐10‐72‐1‐20). The study was registered at the Central Jutland Data Inspectorate (699864). All patients provided written informed consent to participate in the data collection and allowed relevant information in the medical record to be obtained and extracted in concordance with the aim of the study.

## AUTHOR CONTRIBUTIONS

SLS, JHJ, SBM, LS and IM designed the trial. JHJ contributed to the collection of the data. SLS and SBM performed the statistical analyses. SLS wrote the first draft, and JHJ, SBM, LS and IM revised the manuscript. All authors approved the final version.

## Supporting information


**Table S1.** Demographic and clinical characteristics of 334 patients included in the study (differences between patients with complete and incomplete data)

## Data Availability

Research data are not shared.
