# Supplementary material for: Risk stratification for post‐operative pulmonary complications following major cardiothoracic or abdominal surgery: Validation of the PPC Risk Prediction Score for physiotherapist's clinical decision‐making
Source: Clin Respir J. 2023 Jan 3;17(3):229–40. doi: 10.1111/crj.13579 (PMC9978899; doi:10.1111/crj.13579)
Supplement: Supplementary file 1 — Table S1. Demographic and clinical characteristics of 334 patients included in the study (differences between patients with complete and incomplete data) [file CRJ-17-229-s001.docx]

***Supplementary material:***

| Demographic and clinical characteristics of 334 patients included in the study (differences between patients with complete and incomplete data) | | | |  |
| --- | --- | --- | --- | --- |
|  |  |  | **P-value** | |
| **Gender, n (%)**  Female  Male |  |  | 0.449 | |
| **Age, n (%)**  > 80 years  ≤ 80 years |  |  | 0.625 | |
| **Current smoking, n (%)**  Yes  No |  |  | 0.375 | |
| **Chronic productive cough, n (%)**  Yes  No |  |  | 0.046* | |
| **Repeated lung infections, n (%)**  Yes  No |  |  | 0.663 | |
| **Preoperative spirometry, n (%)**  FEV1 ≥ 75 % predicted  FEV1 >50 to < 75 % predicted  FEV1 < 50 % predicted |  |  | 0.985 | |
| **Preoperative oxygen saturation (SpO2)** |  |  | 0.087 | |
| **Unintended weight loss within the last 3 months > 10 kg, n (%)**  Yes  No |  |  | 0.625 | |
| **New mobility score, n (%)**  0-5  6-9 |  |  | <0.001* | |
| **30- second sit to stand test (30CTS), median (IQR) range 5% 95%** |  |  | 0.24 | |
| **Thoracotomy**  Yes  No |  |  | 0.281 | |
| **Laparotomy (aortic)**  Yes  No |  |  | 0.317 | |
| **Laparotomy (gastro-intestinal + urological)**  Yes  No |  |  | 0.551 | |
| **Sternotomy**  Yes  No |  |  | 0.208 | |
| **Hemi-sternotomy**  Yes  No |  |  | 0.867 | |
| **Thoraco-abdominal incision**  Yes  No |  |  | 0.869 | |
| **VATS**  Yes  No |  |  | 0.054 | |
| **PPC (%)**  Yes  No |  |  | 0.795 | |

*Abbreviations: PPC, postoperative pulmonary complications; FEV1, forced expiratory volume in the first second; NYHA, New York, VATS, video assisted thoracoscopic surgery; Heart Association Functional Classification, n, number; IQR, inter quartile range; SD, standard deviation, *statistically significant difference*
